# Fine Mapping Using Whole-Genome Sequencing Confirms Anti-Müllerian Hormone as a Major Gene for Sex Determination in Farmed Nile Tilapia (*Oreochromis niloticus* L.)

**DOI:** 10.1534/g3.119.400297

**Published:** 2019-08-15

**Authors:** Giovanna Cáceres, María E. López, María I. Cádiz, Grazyella M. Yoshida, Ana Jedlicki, Ricardo Palma-Véjares, Dante Travisany, Diego Díaz-Domínguez, Alejandro Maass, Jean P. Lhorente, Jose Soto, Diego Salas, José M. Yáñez

**Affiliations:** * Facultad de Ciencias Veterinarias y Pecuarias, Universidad de Chile, Santiago, Chile,; ** Centro para la Regulación del Genoma, and; †† Centro de Modelamiento Matemático UMI CNRS 2807, Universidad de Chile, Santiago, Chile,; † Programa de Doctorado en Ciencias Silvoagropecuarias y Veterinarias, Campus Sur, Universidad de Chile, Santa Rosa 11315, La Pintana, Santiago, Chile,; ‡ Department of Animal Breeding and Genetics, Swedish University of Agricultural Sciences, Uppsala, Sweden,; § Benchmark Genetics Chile, Puerto Montt, Chile,; ‡‡ Grupo Acuacorporación Internacional (GACI), Cañas, Costa Rica, and; §§ Núcleo Milenio INVASAL, Concepción, Chile

**Keywords:** anti-Müllerian, genome-wide association, quantitative trait loci, sex control, tilapia, whole genome sequencing, Genetics of Sex

## Abstract

Nile tilapia (*Oreochromis niloticus*) is one of the most cultivated and economically important species in world aquaculture. Intensive production promotes the use of monosex animals, due to an important dimorphism that favors male growth. Currently, the main mechanism to obtain all-male populations is the use of hormones in feeding during larval and fry phases. Identifying genomic regions associated with sex determination in Nile tilapia is a research topic of great interest. The objective of this study was to identify genomic variants associated with sex determination in three commercial populations of Nile tilapia. Whole-genome sequencing of 326 individuals was performed, and a total of 2.4 million high-quality bi-allelic single nucleotide polymorphisms (SNPs) were identified after quality control. A genome-wide association study (GWAS) was conducted to identify markers associated with the binary sex trait (males = 1; females = 0). A mixed logistic regression GWAS model was fitted and a genome-wide significant signal comprising 36 SNPs, spanning a genomic region of 536 kb in chromosome 23 was identified. Ten out of these 36 genetic variants intercept the anti-Müllerian (*Amh*) hormone gene. Other significant SNPs were located in the neighboring *Amh* gene region. This gene has been strongly associated with sex determination in several vertebrate species, playing an essential role in the differentiation of male and female reproductive tissue in early stages of development. This finding provides useful information to better understand the genetic mechanisms underlying sex determination in Nile tilapia.

In aquaculture, many fish species of commercial interest exhibit sexual dimorphism in a variety of economically important traits such as growth rate, age at sexual maturity and carcass quality traits (Martínez *et al.* 2014; Purcell *et al.* 2018). When the sexual dimorphism is relevant for production, the identification of genomic regions or markers associated with these traits is of great interest to develop more efficient methodologies for generating a monosex population. Currently, in Nile tilapia the methods for the production of monosex (all-male) populations rely on the use of hormones (Baroiller and D’cotta 2001; Beardmore *et al.* 2001; El-Sayed *et al.* 2012; Alcántar *et al.* 2014).

Teleost fish have developed a variety of sex determination mechanisms, including: i) strict control of genetic factors, ii) control by environmental factors only, or iii) interactions between both genetic and environmental factors (Baroiller *et al.* 2009; Kijas *et al.* 2018). Genetic sex-determining systems may be chromosomal, which involve one gene or master region involved in sex determination, or polygenic, involving several genes or multiple genomic regions (Martínez *et al.* 2014; Palaiokostas *et al.* 2015). Genomic regions associated with sex determination have been identified in some aquaculture species, including chinook salmon (*Oncorhynchus tshawytscha*) (Devlin *et al.* 2001), rainbow trout (*Oncorhynchus mykiss*) (Felip *et al.* 2005), yellow catfish (*Pelteobagrus fulvidraco*) (Wang *et al.* 2009), and Atlantic salmon (*Salmo salar*) (Kijas *et al.* 2018). In recent years, at least six genes have been identified as key factors in the gonadal differentiation pathway. For instance, in medaka species it has been described that the *Dmy* gene regulates sexual differentiation in *Oryzias latipes* (Matsuda *et al.* 2002), *Sox3* gene in *Oryzias dancena* (Takehana *et al.* 2014), and *Gsdf Y* in *Oryzias luzonensis* (Myosho *et al.* 2012). In other teleost fish the genes that regulate sex differentiation are the *Amhy* gene in Patagonian silverside (*Odontesthes hatcheri*) (Hattori *et al.* 2012), *Hsd17b1* gene in fishes of the genus *Seriola* (Purcell *et al.* 2018; Koyama *et al.* 2019), and *sdy - irf9* in rainbow trout (*Oncorhynchus mykiss*) (Yano *et al.* 2012a). The first five genes are implicated in the signaling pathways for sexual differentiation of vertebrates (Herpin and Schartl 2015; Pan *et al.* 2016). The *sdY* gene described for rainbow trout has been proposed as the master gene for sex differentiation in salmonids, which evolved from the immune system-related *irf9* gene and participates in the modulation of the interferon-9 signaling pathway (Yano *et al.* 2012b; Pan *et al.* 2016).

In tilapia, it is suggested that the genetic control of sex is determined primarily by two systems that coexist in the same genus. Nile tilapia (*Oreochromis niloticus*) and Mozambique tilapia (*Oreochromis mossambicus*) have a heterogeneous XX/XY male system (Beardmore *et al.* 2001; Cnaani *et al.* 2008), while blue tilapia (*Oreochromis aureus*) have a heterogeneous ZZ/ZW female system (Cnaani *et al.* 2008). However, other genetic factors and environmental variables, such as temperature may intervene with sex determination (Baroiller and D’cotta 2001; Cnaani *et al.* 2008; Palaiokostas *et al.* 2013, Wessels *et al.* 2014; Eshel *et al.* 2014). To date, different sex-linked genomic regions have been identified in Nile tilapia, including associated regions in linkage groups (LG) 1, 3, 20, and 23 (Lee *et al.* 2003, 2005; Shirak *et al.* 2006; Eshel *et al.* 2010; Cnaani 2013; Palaiokostas *et al.* 2015; Baroiller and D’Cotta 2019). Some studies using populations originated from Egypt (Lake Manzala) and Ghana reported that the sex-determining region is associated to LG1 (Lee *et al.* 2003; Ezaz *et al.* 2004; Lee *et al.* 2005; Lee *et al.* 2011; Palaiokostas *et al.* 2013; Gammerdinger *et al.* 2014; Palaiokostas *et al.* 2015). The presence of genes involved in the cascade of sexual differentiation of vertebrates have been described and mapped in this region, including Wilms tumor suppressor protein 1b (*wt1b*) and cytochrome P450 family 19 subfamilies A member 1 (*cyp19a*) (Lee and Kocher 2007). The *cyp19a* is a strong candidate for involvement in sex determination, as its final product is the aromatase enzyme, which plays a crucial role in ovarian differentiation in vertebrates (Herpin and Schartl 2015; Ma *et al.* 2016).

Through quantitative trait loci (QTL) mapping Eshel *et al.* (2010, 2012) identified a sex-determining region in LG23, which hosts the anti-Müllerian hormone (*Amh*) and the doublesex- and mab-3 related transcription factor 2 (*Dmrt2*) genes (Shirak *et al.* 2006). The *Amh* gene is the mediator of the regression of Müller’s ducts in mammals, birds, and reptiles (Rehman *et al.* 2017). Müller’s ducts are responsible for the development of the uterus and fallopian tubes in females during fetal development (Jamin *et al.* 2003; Pfennig *et al.* 2015). *Dmrt2* gene is a member of the *Dmrt* family of transcription factors suggested to be an essential regulator of male development in vertebrates (Herpin and Schartl 2015). In addition, Sun *et al.* (2014) and Li *et al.* (2015) identified a sex-associated region in LG23 of Nile tilapia. In this region they detected insertions and deletions around and within the *Amh* gene, which were sex-specific (Baroiller and D’Cotta 2019).

The multiple sex-determining regions described for Nile tilapia support the evidence that sex determination is a complex trait, and it is not yet clear which specific putative causative variants are involved in regulating the trait in this species. Previous studies have reported that the strain factor may influence the detection of different genetic regions associated with sex determination in this species (Baroiller and D’Cotta 2019). In this study, we aimed to perform a genome-wide association analysis for sex determination using whole-genome resequencing data obtained from individuals from three different commercial Nile tilapia populations.

## Material and Methods

### Fish

For the present study we used individuals from three commercial breeding populations established in Latin America, which are directly or indirectly related to Genetically Improved Farmed Tilapia (GIFT); the most widely cultivated Nile tilapia strain in the world. The GIFT strain was initially established in the Philippines in 1980 by the World Fish Center to initiate the first breeding program in Nile tilapia. The strain was bred using crosses of four Asian cultured strains from Israel, Singapore, Taiwan and Thailand and four strains from wild populations captured across the natural distribution of this species in Africa (Egypt, Senegal, Kenya, and Ghana) (Neira *et al.* 2016).

We used 59 samples from the POP_A breeding population from AquaAmerica (Brazil), and 126 and 141 samples from POP_B and POP_C breeding populations, respectively, both from Acuacorporación Internacional (ACI, Costa Rica). POP_A was formed from a Malaysian breeding population introduced to Brazil in 2005 for breeding and production purposes. POP_B was generated using individuals from Israel, Singapore, Taiwan and Thailand present in the Philippines in the late 1980s. POP_B strain was imported to Costa Rica by ACI in 2005 from the aquaculture station Carmen Aquafarm (Philippines). POP_C was established in the Philippines by breeding the best available stock from GIFT populations with two of the original African strains that founded GIFT. The detailed origin of these populations were previously described by Yoshida *et al*. (2019).

### DNA extraction and whole-genome sequencing

Genomic DNA was extracted from a total of 326 fish samples, using the Wizard Genomic DNA purification kit (Promega) according to manufacturer’s specifications. DNA quality was evaluated by agarose gel electrophoresis and quantified by a Qubit fluorimeter (Thermo Scientific, USA). After normalization, the sequencing libraries were prepared and barcoded with the 200-cycle DNA PCR-free TruSeq kit in pair-end format and sequenced through 66 lanes of an Illumina HiSeq 2500 machine (Illumina, USA) by a commercial supplier.

### Variant discovery and filtering of SNPs

The sequencing reads of each sample were quality controlled using FASTQC (Andrews 2014) and then aligned using the assembly ASM185804v2 (GenBank accession GCF_001858045.1) of the *O. niloticus* as reference genome (Conte *et al.* 2017), using the BWA mem tool (Li and Durbin 2009; Li and Durbin 2010). The BAM files generated using BWA were further processed with the GATK pipeline (https://www.broadinstitute.org/gatk/) (McKenna *et al.* 2010) in order to get the set of raw SNPs.

Raw SNPs were filtered using Vcftools software v. 0.1.15 (Danecek *et al.* 2011). All INDELs were removed and SNPs were discarded using the following criteria: (1) quality of phred score <40, and (2) non-biallelic SNPs. Additionally, the following filters were applied using the GenAbel R package (Aulchenko *et al.*, 2007): (1) minor allele frequency (MAF) < 0.05, (2) Hardy-Weinberg equilibrium (HWE) p-value < 1x 10^−9^ and, (3) SNP call rate < 0.90. Finally, samples with more than 80% of missing genotypes were also discarded. The genomic regions containing the filtered SNPs were remapped to the actual reference assembly (GenBank accession GCF_001858045.2), using the protocol provided in procedure described by Yáñez *et al.* (2019).

### Basic population genetic statistics and differentiation

The population genetic diversity and differentiation was investigated among the three populations. First, genetic differentiation between populations was measured with pairwise F_ST_ (Weir & Cockerham’S F_ST_) estimated using Vcftools software v. 0.1.15 (Denecek *et al.* 2011). Second, a principal component analysis (PCA) was carried out using PLINK v1.9 (Purcell *et al.* 2007). Finally, the nucleotide diversity was estimated using 20 kb genomic bins and 10 kb step window (–window-pi 20000–window-pi-step 10000) in Vcftools. Genetic differentiation between male and female subpopulations was analyzed using F_ST_ estimates throughout the genome and within the region involved in sex determination. The heterozygosity of each SNP in the sex-associated region was assessed using PLINK v1.9 (Purcell *et al.* 2007). We also estimated the intrapopulation fixation index (F_IS_) for SNPs that were found to be significantly associated with sex determination using software Vcftools.

### Genome-wide association study

GWAS was conducted using the GenAbel R package (Aulchenko *et al.* 2007). The phenotype for sex determination was recorded as “0” for female and “1” for male. To identify the association between SNPs and the sex-determining region in Nile tilapia, a mixed logistic regression model was used, accounting for the binary nature of the sex trait (male/female). A genomic kinship matrix was calculated from SNP data using the *gkin* function. The general formula used for the logistic regression model is as follows:

$$\pi \left(x\right)=\;\frac{e^{\beta_{0}\;+\;\beta_{1\;}*\;SNP\;+\;\beta_{2}*S}}{1+e^{\beta_{0}\;+\;\beta_{1\;}*\;SNP\;+\;\beta_{2}*S}}$$

where π(x) corresponds to the probability that the dependent variable is male (phenotype 1), β0 is the intercept, β1*SNP is the SNP effect and β2*S is the effect of the Nile tilapia strain (with three levels). The -log10 (p-value) for each SNP across the genome was plotted to summarize the GWAS results. The significance threshold was determined by Bonferroni correction (0.05/N, where N is the number of total markers analyzed in the GWAS). The proportion of heritability explained by each significant marker was obtained by comparing estimated heritability with polygenic function and estimated heritability with the inclusion of the significant SNP genotype as a factor (Korte and Farlow 2013).

### SNP Annotation in QTL Region

SNPs located in genomic regions that showed a significant association with sex determination were annotated and categorized to predict the effects of each genetic variant along with the identification of potential candidate genes using the SNPeff software (Cingolani *et al.* 2012).

### Data Availability

Data of genotypes and phenotypes used for this study are available in the online digital repository Figshare https://doi.org/10.6084/m9.figshare.8247050.v4. Furthermore, Supplementary Figure (Figure S1) is also available in Figshare https://doi.org/10.6084/m9.figshare.9178082.v2.

## Results

### Quality control

The whole-genome sequencing (WGS) and posterior alignment of the 326 fish generated an average of 76.9 million raw reads (SD = 65.0 million reads) and 76.3 million mapped reads (SD = 64.6 million mapped reads). The average coverage for each individual was 8.7X (SD = 8.9 X), with a minimum and maximum of 2.1x and 65.7x coverage per fish, respectively. In the discovery phase a total of 38,454,943 genetic variants were identified in the 326 animals. After the quality control steps, which included discarding indels, low-quality variants, exclusion of SNPs other than biallelic and genotypes called below 50% across all individuals, a total of 4,762,199 SNPs were retained. After QC including MAF, HWE, SNPs and sample call rate filtering, a total of 2.3 million high-quality bi-allelic SNPs were retained in 302 samples (144 females and 158 males). Samples that passed the quality control by population were 56 (32 females and 24 males), 114 (42 females and 72 males) and 132 (70 females and 62 males) for POP_A, POP_B and POP_C respectively.

### Basic population statistics and genetic structure

**Figure 1:**
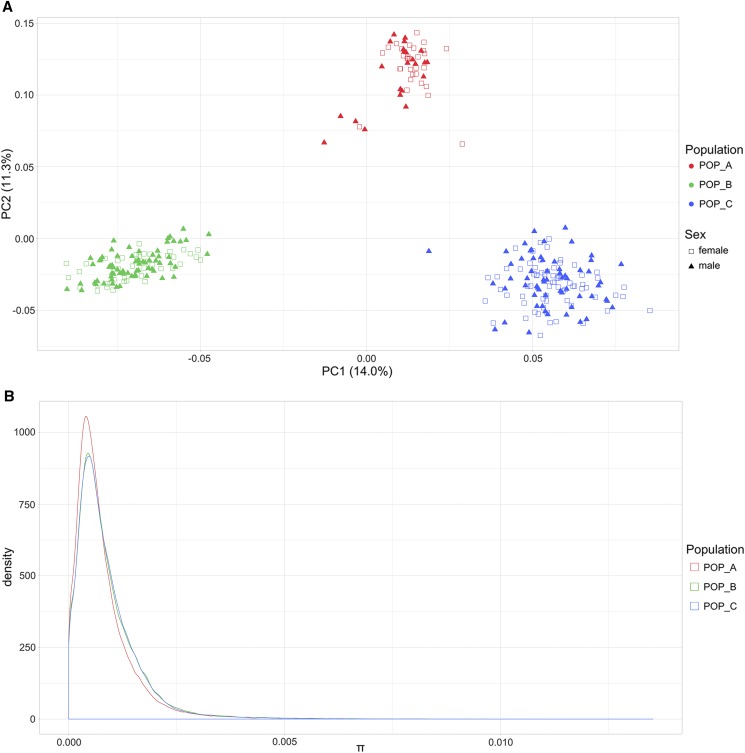
Population genetic structure of males and females, and nucleotide diversity from three Nile tilapia (*Oreochromis niloticus*) farmed populations. (A) Principal Components Analysis for POP_A (red), POP_B (green) and POP_C (blue). Females are represented by unfilled squares and males by triangles. (B) Nucleotide diversity of POP_A (red line), POP_B (green line) and POP_C (blue line).

The population genetic structure was explored through Principal Component Analysis (PCA). The first two principal components represented 25% of the genetic variation. A clear differentiation is observed among the three populations analyzed in this study (Figure 1A). PCA1 allowed us to distinguish the populations POP_B and POP_C and suggested a higher genetic variation for POP_C. In contrast, PCA2 separated the population from Brazil from both populations from Costa Rica; showing a higher variation for POP_A. The global F_ST_ among the three commercial populations was 0.049. The lowest genetic differentiation was observed between POP_B and POP_C (F_ST_ 0.044). The analysis of nucleotide diversity among the populations suggested that POP_A (average π = 7.93 × 10-4) is slightly less diverse than populations from Costa Rica (POP_B average π = 8.78 × 10-4; POP_C π = 8.71 × 10-4) (Figure 1B).

### Genome-wide association study

**Figure 2:**
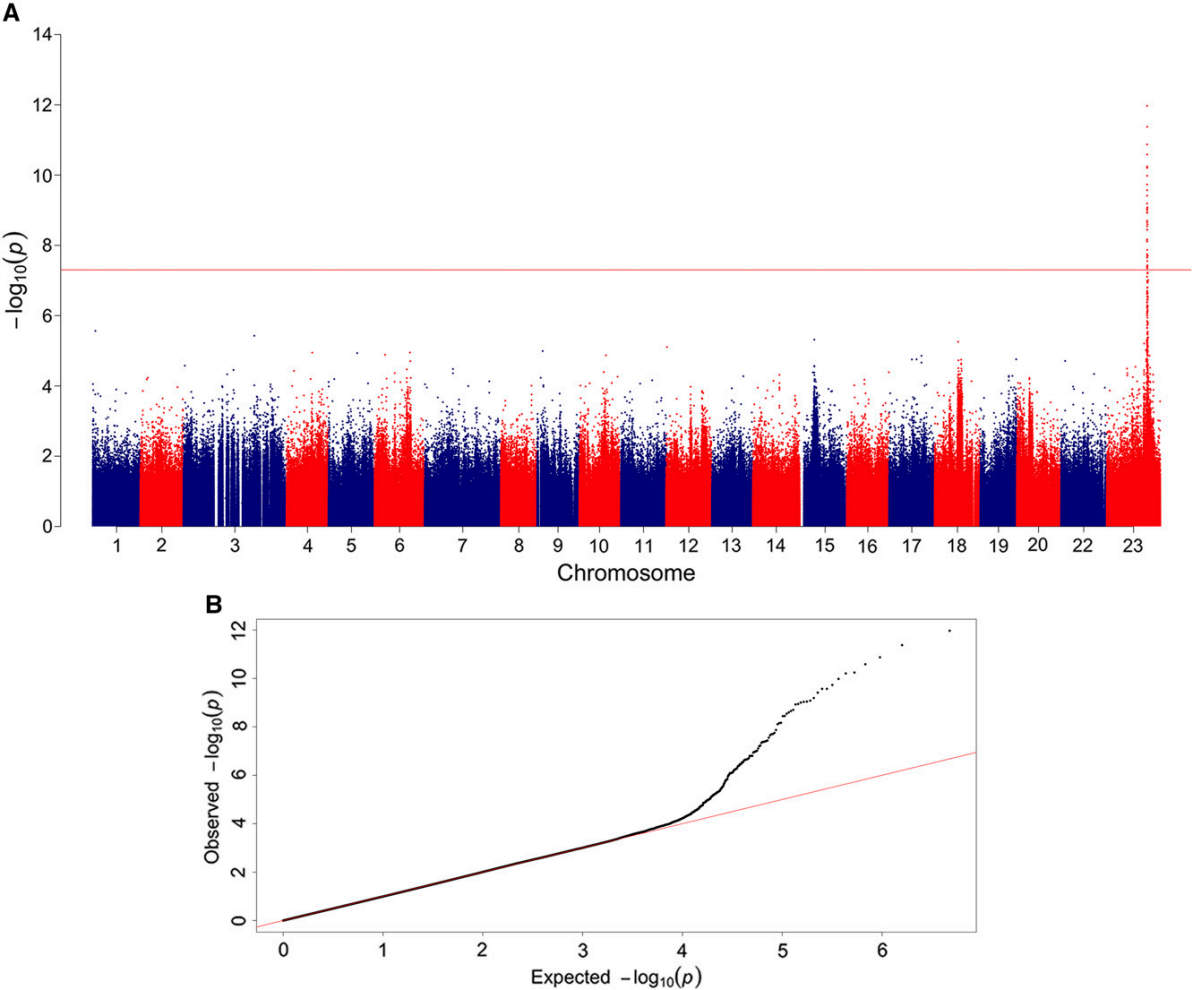
Manhattan plot for GWAS results for sex determination measured as a binary trait (male/female) in Nile tilapia (*Oreochromis niloticus*). (A) The red line indicates the Bonferroni corrected threshold for genome-wide significance. Evidence for genome-wide significant association involving 36 SNPs on chromosome 23. (B) The QQ-plot graph shows the relationship of the theoretical quantiles of the probability distributions between the expected (x-axis) and observed (y-axis) −log_10_(p-values) plotted for each SNP associated with sex determination in Nile tilapia (dots) and the null hypothesis of no association (diagonal solid line).

A genome-wide significant association signal was detected in a genomic region within chromosome 23 (see Figure 2). The genomic region strongly associated with phenotypic sex was comprised of 36 genome-wide significant SNPs. These SNPs were located in a single genomic region which spanned ∼536 kb in linkage group 23 (Table 1). The proportion of the genetic variance explained for phenotypic sex ranged from 0.4 to 0.7 for the significantly associated SNPs (Table 1).

**Table 1:** SNP and genes significantly associated with phenotypic sex in Nile tilapia (*Oreochromis niloticus*)

SNP	LG^1^	Position (bp)	Binary sex	PVAR^3^	Gene
			(p-val^2^)		
**NC_031986.2:34501082**	LG23	34501082	3.03e-13	0.691	*Amh^4^*
**NC_031986.2:34502865**	LG23	34502865	1.28e-12	0.656	*-*
**NC_031986.2:34502864**	LG23	34502864	4.32e-12	0.625	*-*
**NC_031986.2:34510529**	LG23	34510529	8.98e-12	0.619	*-*
**NC_031986.2:34503003**	LG23	34503003	2.15e-11	0.596	*-*
**NC_031986.2:34502748**	LG23	34502748	2.43e-11	0.578	*Amh*
**NC_031986.2:34502756**	LG23	34502756	3.79e-11	0.573	*Amh*
**NC_031986.2:34491574**	LG23	34491574	6.54e-11	0.578	*-*
**NC_031986.2:34503006**	LG23	34503006	1.08e-10	0.550	*-*
**NC_031986.2:34512000**	LG23	34512000	1.38e-10	0.557	*-*
**NC_031986.2:34492336**	LG23	34492336	1.46e-10	0.540	*-*
**NC_031986.2:34500954**	LG23	34500954	2.65e-10	0.524	*Amh*
**NC_031986.2:34492141**	LG23	34492141	3.29e-10	0.512	*-*
**NC_031986.2:34689299**	LG23	34689299	3.43e-10	0.537	*-*
**NC_031986.2:34510584**	LG23	34510584	3.88e-10	0.517	*-*
**NC_031986.2:34509215**	LG23	34509215	4.03e-10	0.531	*-*
**NC_031986.2:34500823**	LG23	34500823	4.61e-10	0.507	*Amh*
**NC_031986.2:34501194**	LG23	34501194	5.84e-10	0.500	*Amh*
**NC_031986.2:34501574**	LG23	34501574	7.71e-10	0.489	*Amh*
**NC_031986.2:34502034**	LG23	34502034	8.58e-10	0.498	*Amh*
**NC_031986.2:34491477**	LG23	34491477	1.18e-09	0.485	*-*
**NC_031986.2:34787936**	LG23	34787936	1.21e-09	0.496	*-*
**NC_031986.2:34502582**	LG23	34502582	1.53e-09	0.477	*Amh*
**NC_031986.2:34500543**	LG23	34500543	1.58e-09	0.486	*Amh*
**NC_031986.2:34433907**	LG23	34433907	3.42e-09	0.469	*Pias4^5^*
**NC_031986.2:34510652**	LG23	34510652	3.64e-09	0.453	—
**NC_031986.2:34504122**	LG23	34504122	4.28e-09	0.467	—
**NC_031986.2:34505919**	LG23	34505919	6.91e-09	0.452	—
**NC_031986.2:34504349**	LG23	34504349	1.00e-08	0.444	—
**NC_031986.2:34503967**	LG23	34503967	1.08e-08	0.443	—
**NC_031986.2:34510699**	LG23	34510699	1.10e-08	0.428	—
**NC_031986.2:34969977**	LG23	34969977	1.48e-08	0.421	—
**NC_031986.2:34642986**	LG23	34642986	1.50e-08	0.419	—
**NC_031986.2:34596057**	LG23	34596057	1.60e-08	0.438	—
**NC_031986.2:34590018**	LG23	34590018	1.83e-08	0.419	*ELL^6^*
**NC_031986.2:34502993**	LG23	34502993	2.07e-08	0.413	—

1 Linkage group.

2 P-value.

3 Proportion of the genetic variance explained by SNP.

4 Anti-Müllerian hormone.

5 Protein inhibitor of activated STAT 4.

6 Elongation factor for RNA polymerase II.

The genomic region of ∼536 kb in the linkage group harboring SNPs significantly associated with phenotypic sex contains about 30 annotated genes. Some of them are strong candidates for having a role in sex determination in Nile tilapia. More interesting, is that ten out of the 36 significantly associated SNPs are located within the anti-Müllerian hormone gene (*Amh*), suggested to be linked to the differentiation process of male and female reproductive tissue in early stages of development of vertebrates and various fish species (Shirak *et al.* 2006; Hattori *et al.* 2012; Pan *et al.* 2016). Moreover, SNPs NC_031986.2:34500823, NC_031986.2:34500954 and NC_031986.2:34501082 are found in a region of the second exon of the *Amh* gene. Markers NC_031986.2:34502582 and NC_031986.2:34502748 also intercept the anti-Mullerian hormone gene in exon 7, while NC_031986.2:34502034 does so in exon 5 of the *Amh* gene. The SNP NC_031986.2:34590018 is found in an intronic region of the Elongation Factor *ELL* (Eleven-Nineteen Lysine-Rich Leukemia), which has been described as a selective co-regulator for steroid receptor functions (Pascual-Le Tallec *et al.* 2005; Zhou *et al.* 2009). SNP NC_031986.2:34433907 is found downstream of the *Pias4* gene, a protein inhibitor of activated STAT (signal transducer and activator of transcription), which inhibits the LRH-1 receptor (liver receptor homolog-1), a gene abundantly expressed in the ovary and shown to activate the transcription of steroid genes, including *Cyp11a1* in granulose cells (Hsieh *et al.* 2009).

### SNP Annotation in QTL Region

**Figure 3:**
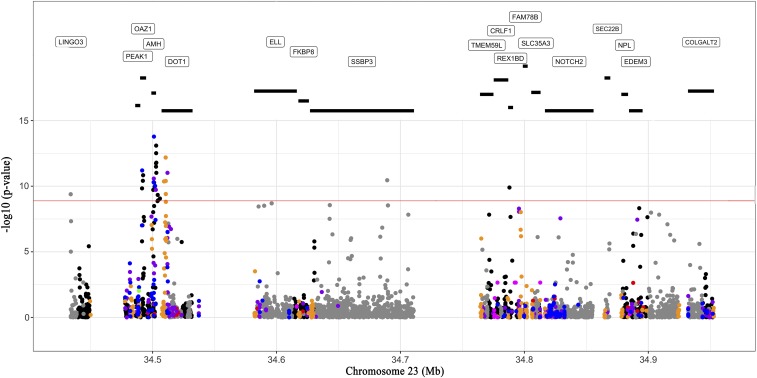
Annotation of the QTL region on linkage group 23. Annotation of the region comprising the 36 SNPs significantly associated with sex determination located in the linkage group 23. The black lines under the genes indicate the dimensions of each gene. The functional annotation of each SNP in this region is as follows: UTR variant (orange dots), Stop gain mutations (green dots), Missense variant (blue dots), 5 UTR premature start codon gain variant (red dots), Intergenic variant (black dots), Intron variant (gray dots), Synonymous variant (purple dots), and Splice region variant (dark pink dots). The red line indicates the Bonferroni corrected threshold for genome-wide significance.

The functional annotation of variants found within the region harbouring SNPs highly associated with sex determination identified the *Amh* gene, which has been suggested to be involved in sex differentiation in the Japanese and Swansea strain of Nile tilapia (Eshel *et al.* 2012; Li *et al.* 2015; Baroiller and D’Cotta 2019). In addition, a SNP was identified as encoding an alleged premature stop codon on the *Amh* gene, while SNPs intercepting the exon regions of *Amh* are synonymous variants. We identified missense variants in other genes flanking the anti-Mullerian hormone gene (Figure 3). Interestingly in the *Amh* intron IV, we identified a SNP that codes for alternative splicing variants. It has been reported that splicing mechanism is especially common in gonads to regulate the activity and function of genes. The alternative splicing can lead to changes in the encoded protein that can influence in the transduction of signals and at the same time affect the correct activation of the receptor-ligand complex in these proteins (Pfennig *et al.* 2015). In European sea bass (*Dicentrachus labrax*) two sex-specific alternative splicings have been reported for the *Amh* gene (Halm *et al.* 2007), and in Barramundi (*Lates calcarifer*), two concurrent sex-specific alternative splicing forms were identified within the *dmrt1* and *cyp19a1* genes (Domingos *et al.* 2018).

### Genetic and heterozygosity differentiation between males and females

**Figure 4:**
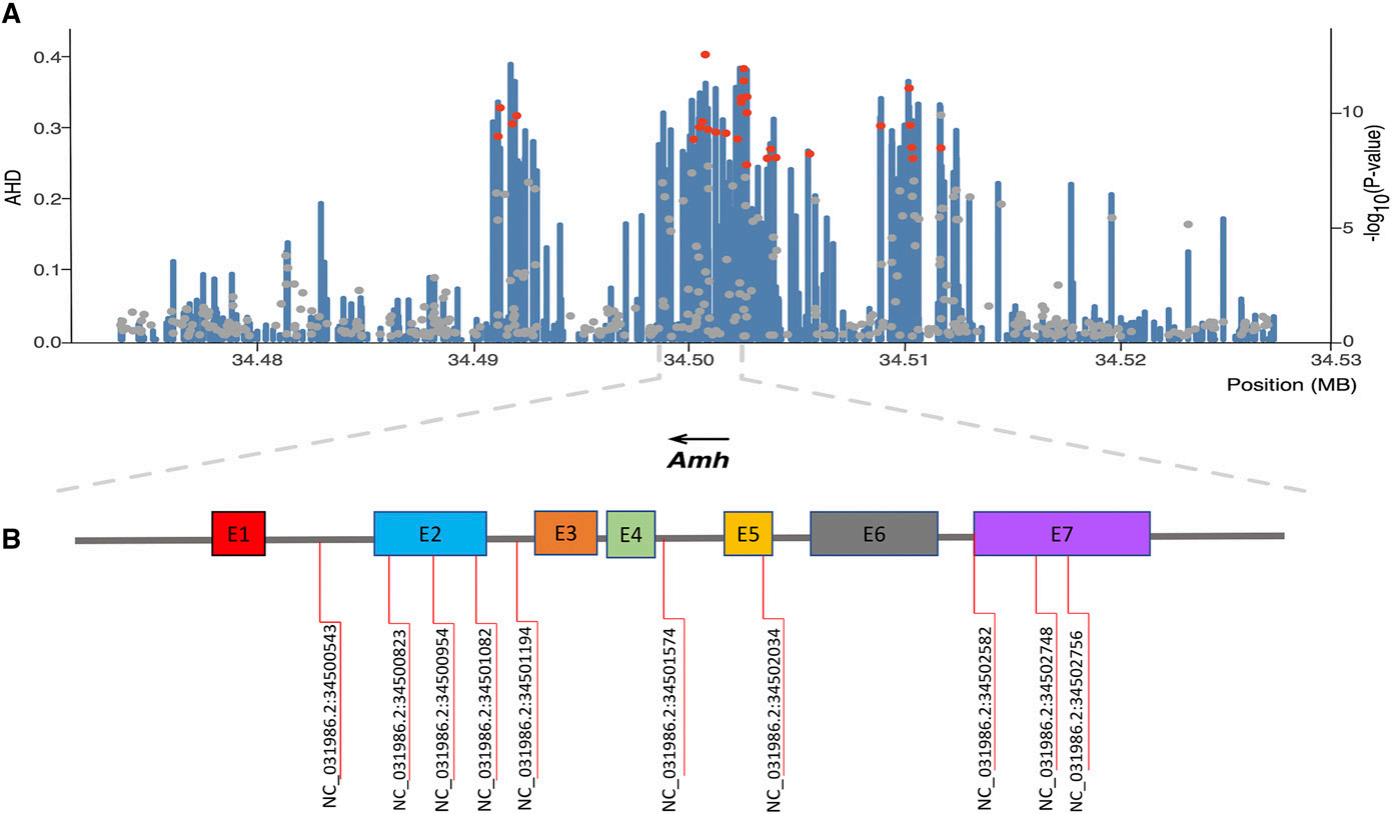
Regional plot of SNP associated with sex determination on chromosome 23. (A) SNPs are plotted by position on the chromosome (x-axis) and the average heterozygosity difference (AHD) between males and females across all populations is represented by blue bars (y-axis). The significance (-log10(p-value)) of SNPs associated with phenotypic sex (red dots) and harboring the *Amh* gene (gray dots) is also shown (secondary y-axis). (B) The *Amh* gene and SNPs significantly associated with sex determination.

The overall F_ST_ estimate between males and females across all populations was 0.0003, indicating a lower genetic differentiation between male and female sub-populations. In the 536 Kb genomic region associated with the sex determination in chromosome 23, the average F_ST_ was 0.004 (Figure S1). These results suggest that there is a higher degree of genetic differentiation between male and female sub-populations in this genomic region compared to the average genetic differentiation across the whole genome. In the same genomic region across the 36 significant SNPs, the average of F_IS_ for males was -0.08102, while for females was 0.5184, suggesting that there is heterozygote deficit for females in this genomic region compared to males. To search for highly heterozygote loci in males (potentially XY) compared to females (potentially XX), the average heterozygosity differences between males and females were locally calculated for each SNP in the associated region within linkage group 23. Interestingly, we found co-localization of highly heterozygote variants in males when compared to females in the region harboring the most significant SNPs within and near the *Amh* gene (Figure 4).

## Discussion

The main objective of the present study was to identify genomic regions involved in sex determination in Nile tilapia using genome-wide association analysis based on whole-genome sequencing of fish from different commercial populations. Previous studies have emphasized on the complexity of determining and identifying genomic regions associated with sex determination in Nile tilapia. For instance, the markers reported to be associated with phenotypic sex have been located in different linkage groups including LG1, LG3, LG20, and LG23 (Lee *et al.*, 2003; Ezaz *et al.* 2004; Lee *et al.* 2011; Eshel *et al.* 2010; Eshel *et al.* 2012; Palaiokostas *et al.* 2015). Palaiokostas *et al.* (2013) suggested that one of the main limitations to detect sex-determining regions in *O. niloticus* was the limited number of genetic markers avaliable. Additionally, Baroiller and D’Cotta (2019) suggested that different regions involved in sex determination can be found when different strains or hybrids are used, *e.g.* LG1 was identified as sex determinant for strains originated from Manzala or Ghana, while LG23 was identified for the Japanese and Swansea strains.

In this study we analyzed three different farmed Nile tilapia populations which show a low level of genetic differentiation based on the global F_ST_ estimate. This low differentiation is probably because the three Nile tilapia populations share common ancestors and are related due to the common origin of the GIFT strain (Yoshida *et al*., 2019). The lowest genetic differentiation was observed between POP_B and POP_C (F_ST_ 0.044), which is concordant with the common geographical origin of these farmed populations (Costa Rica). However, the three populations clustered in clear groups in the PCA, indicating a patent genetic structure among the three populations.

The approach used in this study was based on whole-genome sequencing of males and females from three different farmed populations providing a highly dense distribution of markers and covering the whole genome. The GWAS revealed a single genomic region in linkage group 23 that is associated with sex determination in Nile tilapia. These results are consistent with those previously reported (Shirak *et al.* 2006; Cnaani *et al.* 2008; Lühmann *et al.* 2012; Wessels *et al.* 2014) and suggested that the sex determination region in Nile tilapia would be in linkage group 23. Eshel *et al.* (2010, 2014) described a sex-associated QTL by using microsatellite markers in this linkage group, while Joshi *et al.* (2018), using a panel of 58K SNP markers, reported that the most likely position of the sex locus would be found in the LG23.

We identified 36 significant genome-wide markers associated with sex on chromosome 23. Ten markers intercept the anti-Müllerian hormone gene, which mediates the regression of Müller’s ducts in several vertebrate species (Jamin *et al.* 2003). The Müller ducts are responsible for the development of the uterus and fallopian tubes in females during fetal development (Jamin *et al.* 2003; Pfennig *et al.* 2015). The *Amh* gene has previously been considered a candidate gene for sex determination in Nile tilapia, as suggested by Eshel *et al.* (2012). (Sun *et al*. 2014) Moreover Wessels *et al.* (2014) described a missense SNP in the second exon of *Amh*, associated to temperature-dependent sex reversal and four other SNPs flanking the *Amh* gene (Wessels *et al.* 2017).

Two previous reports have identified a duplicate of the male-specific *Amh* gene in LG23 called *Amhy*, which differs from the *Amh* gene sequence by a deletion of 233 bp in exon VII, hence lacking the capability to encode the protein motif that binds to the transforming growth factor beta receptor (TGF-β domain) (Eshel *et al.* 2014). Li *et al.* (2015), reported a tandem duplication located immediately downstream of *Amh* on the Y chromosome (*Amhy*). The coding sequence was identical to *Amh* linked to the X, except for a missense SNP in exon II, which changes an amino acid in the N-terminal region. Also, *Amhy* lacks 5608 bp of promoter sequence that is found in the X-linked *Amh* homolog.

Our study identified SNPs that intercept the *Amh* gene in different positions, similar to previous studies. For instance, three SNPs located in exon II were identified, while Li *et al.* (2015) reported a missense SNP in exon II in the tandem duplicate of *Amh*. A missense SNP in exon II was also described to be associated with the temperature-dependant sex reversal in Nile tilapia (Wessels *et al.* 2014). Finally Furthermore, two SNPs are located in the coding sequence of exon VII. These findings are consistent with the locations of the variants reported by Eshel *et al.* (2014), for exon VII in the duplicate of the specific *Amh* gene in males.

Ijiri *et al.* (2008) and Eshel *et al.* (2014) detected dimorphic expression of the *Amh* gene during gonadal differentiation and early development in undifferentiated tilapia gonads from 2 days after fertilization. These authors showed that from day 5 after fertilization the expression of the *Amh* gene increases sharply in male gonads until day 35 after fertilization, demonstrating a crucial role of this gene in sexual differentiation. This overexpression of *Amh* in male gonads in the early stages of development has been also reported in Japanese sole (*Paralichthys olivaceus*) (Yoshinaga *et al.* 2004).

The *Amh* gene is a member of the beta-transforming growth factor family (TGFβ). In some species of teleost fish, members of the TGFβ family and steroidogenic genes encoding the enzyme 17β-hydroxysteroid dehydrogenase 1 (*Hsd17b1*) have been identified as sex determination factors, in contrast to other vertebrates where key sex determination switches are transcription factors (Herpin and Schartl 2015; Koyama *et al.* 2019). Hattori *et al.* (2012) demonstrated that a duplicate of the *Amh* gene is found only in males, suggesting that the duplicate gene is responsible for the sex determination in Patagonian silverside (*Odontesthes hatcheri*). In puffer fish (*Takifugu rubripes*), it was determined that the change of an SNP in the anti-Müllerian hormone receptor was associated with sex differentiation (Kamiya *et al.* 2012). Bej *et al.* (2017) identified that the duplicate of the truncated *Amh* gene is found only in males, suggesting that some structural differences are responsible for sex determination in the Old World atheriniform. In *Oryzias luzonensis*, it was reported that the factor derived from gonadal somatic cell derived factor (*Gsdf*), another member of the family TGFβ, is a sex determinant; demonstrating that the expression of *Gsdf* was only present in the male gonads during the gonadal differentiation (Myosho *et al.* 2012). The antecedents reported in other fish species, together with those obtained in this study allow us to suggest that members of the TGFβ family are relevant in the determination of sex in fish species. A missense SNP in the steroidogenic gene *Hsd17b1*, was recently associated with sex differentiation in *Seriola* genus (Koyama *et al.* 2019).

We evaluated genetic differentiation between male and female subpopulations using estimates of F_ST_ throughout the genome and within the region involved in sex determination. The average F_ST_ was 0.004 between male and female in the genomic region of 536 Kb associated with sex determination located on chromosome 23. However, Wessels *et al.* (2017) reported F_ST_ values above 0.3506 in the sex-associated region at LG23 among subpopulations of females and pseudo-males treated at high temperatures. The overall F_ST_ estimate between males and females across all populations was 0.0003, indicating a lower overall genetic differentiation between both sub-populations.

It is interesting to note that seven SNPs that intercept the *Amh* gene account for a relatively high proportion of genetic variance. SNP NC_031986.2:34501082 intercepts the second exon in the *Amh* gene and explains a proportion of 0.69 of the additive genetic variance. In contrast, Palaiokostas *et al.* (2015) using an approach based on ddRADseq to identify genomic regions in animals from Egypt and Ghana have indicated a significant QTL in LG1, which would explain 40.5% of the phenotypic variance. These differences observed in both studies could be influenced by population structure and origin, environmental effects, marker density, and accuracy of trait measurement (Doerge 2002).

This study confirms that the *Amh* gene is an important gene that controls sexual differentiation in Nile tilapia, considering the relatively high proportion of genetic variance explained by the significant markers. The findings obtained in this study can be used as a potential tool to detect genetic sex in farmed populations of Nile tilapia, which in turn can be used to optimize methods for producing all-male populations for production.

### Conclusions

This study provides further evidence to better understand the genetic architecture of sex determination in commercial Nile tilapia populations established in Latin America. Ten SNPs highly associated with sex determination intercept the anti-Müllerian hormone gene (*Amh*) providing strong evidence of *Amh* being a major gene controlling sex differentiation in Nile tilapia farmed populations.
